# Diagnostic value of 3D arterial spin labeling plus diffusion-weighted imaging for transient ischemic attack and short-term stroke prognosis: A retrospective cohort study

**DOI:** 10.1097/MD.0000000000048059

**Published:** 2026-03-20

**Authors:** Yanmei Fu, Tongyu Shang, Peiqi Wang, Chuan Peng

**Affiliations:** a Department of Medical Imaging Center, Affiliated Hospital of Panzhihua University, Panzhihua City, China.

**Keywords:** 3D-ASL, diffusion-weighted imaging, multimodal imaging, prognosis, stroke risk, transient ischemic attack

## Abstract

Transient ischemic attack (TIA) carries substantial short-term stroke risk, yet current diagnostics lack adequate sensitivity. Diffusion-weighted imaging (DWI) detects only 30% to 50% of TIA-related lesions, while 3-dimensional arterial spin labeling (3D-ASL) offers complementary hemodynamic assessment. We performed a retrospective cohort study of 106 TIA patients and 58 healthy controls undergoing 3.0T MRI within 72 hours of symptom onset. Imaging included DWI (b = 0,1000 seconds/mm^2^), 3D-ASL with dual post-labeling delays, and MR angiography. Clinical data and ABCD^2^ scores were collected, with 90-day stroke follow-up. Diagnostic performance and prognostic factors were analyzed using ROC curves and Cox regression. DWI detected acute lesions in 37 patients (34.9%), while 3D-ASL revealed cerebral hypoperfusion in 64 (60.4%), including 35 DWI-negative cases. Combined imaging improved 90-day stroke prediction (AUC 0.854; sensitivity 93.8%, specificity 71.1%) versus DWI alone (AUC 0.761) or 3D-ASL alone (AUC 0.790) (*P* <.05). During follow-up, 16 patients (15.1%) experienced stroke. Multivariate analysis identified DWI positivity (HR 3.24, 95% CI: 1.18–8.91), hypoperfusion volume >15 mL (HR 2.87, 95% CI: 1.06–7.78), and ABCD^2^ score ≥4 (HR 3.01, 95% CI: 1.09–8.31) as independent predictors. Symptom duration ≥1 hour correlated with higher DWI positivity (47.7% vs 25.8%, *P* = .012) and larger hypoperfusion volumes (16.8 vs 9.2 mL, *P* = .004). Combining 3D-ASL with DWI significantly improves cerebrovascular pathology detection and 90-day stroke risk prediction beyond clinical scoring, identifying high-risk TIA patients for intensified secondary prevention.

## 1. Introduction

Transient ischemic attack (TIA) is a well-established critical precursor to ischemic stroke, with reported 90-day stroke risk ranging from 5% to 20% across clinical cohorts.^[1]^ The risk of recurrent cerebrovascular events is highest in the first 7 days post-TIA, making accurate early risk stratification essential for guiding timely secondary prevention and reducing adverse outcomes.^[2]^

Clinically, the ABCD^2^ scoring system is the most widely used tool for TIA risk assessment, as it provides a simple framework to categorize patients into low-, moderate-, and high-risk groups.^[3]^ However, its utility is limited by inherent shortcomings: it relies heavily on clinician judgment to evaluate symptom severity, introducing subjective variability into risk estimates, and its predictive accuracy remains moderate in real-world practice – with the area under the receiver operating characteristic curve (AUC) typically ranging from 0.65 to 0.75.^[4]^ Recent studies highlight this gap, demonstrating that even “low-risk” TIA patients (ABCD^2^ scores 0–3) face 5% to 8% 90-day stroke risk when underlying cerebral hemodynamic impairment exists. This indicates that clinical scoring alone may miss occult high-risk subgroups.^[5]^

To address these limitations, the diagnostic paradigm for TIA has shifted from a time-based definition (<24 hours of symptoms) to a tissue-based criterion, which requires ruling out acute infarction via neuroimaging.^[6]^ Diffusion-weighted imaging (DWI) has become the cornerstone of this paradigm, as it detects acute ischemic lesions in 20% to 50% of clinically defined TIA cases and identifies patients with a 2.5 to 8-fold higher risk of recurrent stroke.^[7,8]^ Yet DWI has notable drawbacks: its sensitivity is low for transient or micro-embolic events (e.g., lesions <3 mm or with reversible cytotoxic edema), and its specificity is compromised by motion artifacts or magnetic susceptibility effects from bone-air interfaces.^[9]^ Consequently, 10% to 15% of DWI-negative TIA patients develop stroke within 90 days, representing a “hidden high-risk” population undetected by conventional imaging.^[10]^

Three-dimensional arterial spin labeling (3D-ASL) offers a noninvasive, contrast-free alternative to complement DWI: unlike DWI, which visualizes irreversible infarction, 3D-ASL quantifies cerebral blood flow (CBF) and detects reversible hypoperfusion in ischemic penumbra or watershed zones – tissue at risk but not yet infarcted.^[11,12]^ Previous studies have demonstrated that 3D-ASL is capable of detecting cerebral hypoperfusion in a substantial proportion of TIA patients with negative DWI findings.^[13]^ However, critical evidence gaps persist: few studies have quantified 3D-ASL-derived parameters (e.g., hypoperfusion volume) to validate their prognostic significance for subsequent stroke; the additive benefit of combining 3D-ASL with DWI (especially alongside ABCD^2^ scoring) is unestablished; and the impact of symptom duration on 3D-ASL/DWI concordance and stroke risk has not been thoroughly explored.

This retrospective cohort study aimed to address these gaps by evaluating the diagnostic value of combined 3D-ASL and DWI for TIA-related cerebrovascular pathology, identifying quantitative 3D-ASL parameters predictive of 90-day stroke, and assessing if integrating 3D-ASL/DWI with ABCD^2^ improves risk stratification beyond single modalities or clinical scoring. We hypothesized that combining 3D-ASL and DWI would enhance TIA pathophysiology detection and risk stratification – especially for DWI-negative patients.

## 2. Methods

### 2.1. Study design and participants

This retrospective cohort study was conducted at our hospital between May 2022 and May 2023. We reviewed electronic medical records of 328 consecutive patients presenting with suspected TIA. After applying exclusion criteria, 106 patients were finally enrolled as the study group. Exclusions were due to: MRI contraindications (n = 67), nonischemic etiologies (n = 48), incomplete imaging data (n = 38), previous stroke history (n = 28), intracranial hemorrhage/tumor (n = 23), and severe organ dysfunction (n = 18).

The inclusion criteria were: age ≥18 years; final diagnosis of TIA according to traditional time-based criteria (focal neurological symptoms lasting <24 hours); completion of brain MRI including DWI and 3D-ASL within 72 hours of symptom onset; available complete clinical data and follow-up records.

Additionally, 58 age- (±5 years) and sex-matched healthy controls were selected from 215 health checkup participants, with no cerebrovascular disease history, no cardiovascular disease (e.g., myocardial infarction) or metabolic disorders (e.g., hypertension, diabetes).

This study was approved by the Institutional Review Board of Affiliated Hospital of Panzhihua University (No. 2025-09-010) and was conducted in full accordance with local legislation and institutional guidelines. The requirement for informed consent was waived because the investigation used de-identified data collected during routine clinical care.

### 2.2. Imaging protocol

All examinations were performed on a 3.0T Siemens Prisma MRI scanner (Siemens Healthineers, Erlangen, Germany) using a 12-channel head coil. The imaging protocol included:

DWI: Single-shot echo-planar imaging (SS-EPI) with b-values of 0 and 1000 seconds/mm^2^. Key parameters: TR = 5000 ms, TE = 83 ms, slice thickness = 3 mm, FOV = 240 × 240 mm^2^. ADC maps were automatically generated via Siemens Syngo.via Version 4.0. ADC reduction rate was calculated as: (ADC of contralateral side - ADC of ipsilateral side)/ADC of contralateral side × 100%. ROIs (≥5 mm^2^) were manually drawn on lesions/contralateral tissue (3 repeats for mean value).

Three-dimensional arterial spin labeling (3D-ASL): Pseudocontinuous ASL (pCASL) with dual post-labeling delays (1.5 and 2.5 seconds). Key parameters: TR = 4600 ms, TE = 10.5 ms, slice thickness = 3 mm. CBF maps were processed via Syngo.via. measured parameters: mean whole-brain CBF; hypoperfusion volume (defined as CBF <30 mL/100g/min; software-segmented with manual adjustment); relative CBF (rCBF = CBF of affected side/CBF of contralateral side, <0.8 = hypoperfusion).

Magnetic resonance angiography (MRA): 3-dimensional time-of-flight (3D-TOF-MRA) was used to evaluate intracranial major vessel stenosis, graded as: Grade 0, no stenosis; Grade 1, <50% stenosis; Grade 2, 50% to 70% stenosis; Grade 3, >70% stenosis.

Two radiologists with 10 years of experience in neuroimaging independently reviewed all images blinded to clinical data. Discrepancies were resolved by consensus with a third senior radiologist. Spatial co-registration between 3D-ASL perfusion maps and DWI images was performed using dedicated software.

### 2.3. Clinical data collection and follow-up

Demographic and clinical variables were extracted from electronic medical records, including age, sex, vascular risk factors (hypertension: BP ≥140/90 mm Hg or antihypertensive use; diabetes: fasting glucose ≥7.0 mmol/L or hypoglycemic use; hyperlipidemia: TC ≥5.2 mmol/L or LDL-C ≥3.4 mmol/L or lipid-lowering use; smoking: current or quit <1 year), symptom duration, TIA symptom type, and ABCD^2^ score components.

Patients were followed up at 30, 60, and 90 days via in-person visits (84.0%) or telephone interviews (16.0%) using a standardized questionnaire. The primary endpoint was new ischemic stroke within 90 days, confirmed by brain MRI within 24 hours and defined per WHO criteria. Follow-up completion rate was 100% (no lost cases).

### 2.4. Statistical analysis

Statistical analysis was performed using SPSS version 26.0 (Chicago). The normality of continuous variables was formally assessed using the Shapiro–Wilk test, which is particularly suitable for small-to-medium sample sizes (n <50). The Levene test was used to evaluate homogeneity of variances. For variables with normal distribution (Shapiro–Wilk *P* >.05) and equal variances (Levene test *P* >.05), independent t-tests were applied; otherwise, Mann–Whitney *U* tests were used for non-normally distributed variables or those with unequal variances. Continuous variables were expressed as mean ± standard deviation or median with interquartile range, and compared using independent *t*-test or Mann–Whitney *U* test. Categorical variables were presented as frequencies and percentages, analyzed by Chi-square test or Fisher exact test.

Diagnostic performance was evaluated by sensitivity, specificity, PPV, NPV, and AUC. The correlation between hypoperfusion volumes on 3D-ASL and DWI-positive lesions was assessed using Pearson or Spearman correlation coefficient. Cox proportional hazards regression was used to identify parameters associated with stroke recurrence. All tests were 2-sided, and *P* <.05 was considered statistically significant.

## 3. Results

### 3.1. Comparison of baseline characteristics between TIA patients and healthy controls

A total of 106 TIA patients (mean age 66.3 ± 8.7 years; 58.5% male) and 58 healthy controls (mean age 65.1 ± 7.9 years; 56.9% male) were included (Table 1). No significant differences were observed in age or gender distribution between groups (*P* >.05). Vascular risk factors were significantly more prevalent in the TIA group: hypertension (71.7% vs 32.8%, *P* <.001), diabetes mellitus (31.1% vs 10.3%, *P* = .003), hyperlipidemia (52.8% vs 24.1%, *P* <.001), and smoking history (41.5% vs 15.5%, *P* <.001). The median ABCD^2^ score was 4 (interquartile range [IQR] 3–5) in TIA patients, with 38.7% classified as high risk (≥4). Symptom duration was <1 hour in 58.5% of patients, 1 to 6 hours in 31.1%, and >6 hours in 10.4%.

**Table 1:** Demographics and clinical characteristics of TIA patients and healthy controls.

Characteristic	TIA group (n = 106)	Control group (n = 58)	*P*-value
Age (yr), mean ± SD	66.3 ± 8.7	65.1 ± 7.9	.384
Male sex, n (%)	62 (58.5)	33 (56.9)	.823
Vascular risk factors			
Hypertension, n (%)	76 (71.7)	19 (32.8)	<.001
Diabetes mellitus, n (%)	33 (31.1)	6 (10.3)	.003
Hyperlipidemia, n (%)	56 (52.8)	14 (24.1)	<.001
Smoking history, n (%)	44 (41.5)	9 (15.5)	<.001
ABCD^2^ score			
Median (IQR)	4 (3–5)	–	–
0-3 (low risk), n (%)	41 (38.7)	–	–
4–5 (moderate risk), n (%)	52 (49.1)	–	–
6–7 (high risk), n (%)	13 (12.3)	–	–
Symptom duration			
<1 h, n (%)	62 (58.5)	–	–
1–6 h, n (%)	33 (31.1)	–	–
>6 h, n (%)	11 (10.4)	–	–

TIA = transient ischemic attack.

Age (Shapiro–Wilk W = 0.984, *P* = .312), ADC reduction rate (W = 0.978, *P* = .421), and whole-brain CBF in controls (W = 0.981, *P* = .287) showed normal distribution and equal variances (Levene test, all *P* >.05), justifying the use of independent *t*-tests. In contrast, hypoperfusion volume (W = 0.843, *P* <.001), symptom duration (W = 0.756, *P* <.001), and ABCD^2^ scores (W = 0.891, *P* = .008) exhibited significant departures from normality, requiring Mann-Whitney U tests for group comparisons.

### 3.2. Multimodal MRI detection rates and quantitative imaging parameters

DWI revealed acute ischemic lesions in 37 patients (34.9%), with a mean ADC reduction rate of 18.6 ± 6.3%. 3D-ASL detected cerebral hypoperfusion (rCBF <0.8) in 64 patients (60.4%), including 28 cases with DWI-negative findings. The median hypoperfusion volume was 12.5 mL (IQR 6.8–21.4). MRA identified intracranial arterial stenosis in 51 patients (48.1%): Grade 1 in 22.6%, Grade 2 in 18.9%, and Grade 3 in 6.6% (Table 2).

**Table 2:** MRI findings in TIA patients.

Imaging modality	Finding	n (%)
DWI	Positive (acute lesion)	37 (34.9)
	Negative	69 (65.1)
3D-ASL	Hypoperfusion (rCBF < 0.8)	64 (60.4)
	Normal perfusion	42 (39.6)
MRA	Grade 0 (no stenosis)	55 (51.9)
	Grade 1 (<50%)	24 (22.6)
	Grade 2 (50%–70%)	20 (18.9)
	Grade 3 (>70%)	7 (6.6)
Combined findings	DWI+/3D-ASL+	29 (27.4)
	DWI+/3D-ASL-	8 (7.5)
	DWI-/3D-ASL+	35 (33.0)
	DWI-/3D-ASL-	34 (32.1)

3D-ASL = 3-dimensional arterial spin labelling, DWI = diffusion-weighted imaging, MRA = magnetic resonance angiography, TIA = transient ischemic attack.

### 3.3. Comparative predictive performance of individual and combined imaging modalities

The combined 3D-ASL and DWI approach demonstrated superior diagnostic accuracy compared with either modality alone (Table 3). For predicting 90-day stroke risk, the sensitivity and specificity were 68.8% and 83.3% for DWI alone, 81.3% and 76.7% for 3D-ASL alone, and 93.8% and 71.1% for combined imaging, respectively. The AUC was 0.761 (95% CI: 0.654–0.868) for DWI, 0.790 (95% CI: 0.689–0.891) for 3D-ASL, and 0.854 (95% CI: 0.771–0.937) for combined assessment. The difference in AUC between combined and single-modality approaches was statistically significant (*P* <.05).

**Table 3:** Predictive performance for 90-d stroke risk.

Imaging strategy	Sensitivity (%)	Specificity (%)	PPV (%)	NPV (%)	AUC (95% CI)	*P*-value*
DWI alone	68.8 (11/16)	83.3 (75/90)	29.7	96.0	0.761 (0.654–0.868)	.041
3D-ASL alone	81.3 (13/16)	76.7 (69/90)	20.3	97.3	0.790 (0.689–0.891)	.028
DWI + 3D-ASL (combined)	93.8 (15/16)	71.1 (64/90)	21.7	99.4	0.854 (0.771–0.937)	Reference

3D-ASL = 3-dimensional arterial spin labelling, AUC = area under the curve, DWI = diffusion-weighted imaging, NPV = negative predictive value, PPV = positive predictive value.

* *P*-value compared with combined approach.

### 3.4. Perfusion-diffusion correlation and impact of imaging concordance on stroke risk

The relationship between perfusion and diffusion parameters, along with 90-day stroke rates stratified by imaging concordance, is presented in Table 4. A significant positive correlation was observed between hypoperfusion volume on 3D-ASL and ADC reduction rate in DWI-positive lesions (*R* = 0.512, *P* = .001).

**Table 4:** Perfusion-diffusion correlation and 90-d stroke risk by imaging concordance.

Analysis Type	Finding	Value/rate	Statistical result
Correlation	Hypoperfusion volume vs. ADC reduction	*R* = 0.512	*P* = .001
Stroke rate by pattern	DWI+/ASL + (n = 29)	31.0% (9/29)	χ^2^ = 8.21, *P* = .002*
	DWI+/ASL- (n = 8)	12.5% (1/8)	χ^2^ = 0.57, *P* = .451*
	DWI-/ASL + (n = 35)	20.0% (7/35)	χ^2^ = 7.12, *P* = .008*
	DWI-/ASL- (n = 34)	0.0% (0/34)	Reference group

ADC = apparent diffusion coefficient, ASL = arterial spin labelling, DWI = diffusion-weighted imaging.

* Compared with DWI-/ASL- group. r: Pearson correlation coefficient.

Stroke risk varied substantially across imaging patterns. Patients with dual-positive findings (DWI+/ASL+) demonstrated the highest stroke rate at 31.0% (9/29), which was significantly higher than the reference group (χ^2^ = 8.21, *P* = .002). The DWI+/ASL- pattern showed an intermediate stroke rate of 12.5% (1/8), though this difference did not reach statistical significance compared with the reference group (χ^2^ = 0.57, *P* = .451). Notably, patients with isolated perfusion abnormalities (DWI-/ASL+) exhibited a significantly elevated stroke rate of 20.0% (7/35) relative to the reference group (χ^2^ = 7.12, *P* = .008). In contrast, patients with concordant negative findings (DWI-/ASL-) experienced no stroke events during follow-up (0/34, 0.0%).

### 3.5. Clinical outcomes at 90 days and multivariate risk factor analysis

During 90-day follow-up, 16 patients (15.1%) experienced new ischemic stroke, 9 (8.5%) had recurrent TIA, 2 (1.9%) died from vascular causes and 22 had composite vascular event (Table 5). Stroke recurrence rates differed significantly by imaging status: 10/37 (27.0%) in DWI-positive patients vs 6/69 (8.7%) in DWI-negative patients (*P* = .008); and 12/64 (18.8%) in 3D-ASL-positive patients vs 4/42 (9.5%) in 3D-ASL-negative patients (*P* = .176).

**Table 5:** Multivariate risk factor analysis of 90-d outcomes.

	Hazard ratio (95% CI)	*P*-value
DWI positive	3.24 (1.18–8.91)	.022
Hypoperfusion volume >15 mL	2.87 (1.06–7.78)	.038
ABCD^2^ score ≥4	3.01 (1.09–8.31)	.033
MRA grade 3 stenosis	2.56 (0.92–7.13)	.071

3D-ASL = 3-dimensional arterial spin labelling, DWI = diffusion-weighted imaging, MRA = magnetic resonance angiography.

Multivariate Cox regression analysis identified 3 independent predictors of 90-day stroke: DWI positivity (HR 3.24, 95% CI: 1.18–8.91, *P* = .022), hypoperfusion volume >15 mL (HR 2.87, 95% CI: 1.06–7.78, *P* = .038), and ABCD^2^ score ≥4 (HR 3.01, 95% CI: 1.09–8.31, *P* = .033). MRA Grade 3 stenosis showed a trend toward significance (HR 2.56, 95% CI: 0.92–7.13, *P* = .071).

### 3.6. Stratified analysis by symptom duration and arterial stenosis severity

Stratified by symptom duration, patients with symptoms ≥1 hour demonstrated higher DWI positivity (48.9% vs 25.8%, *P* = .012) and larger hypoperfusion volumes (median 16.8 vs 9.2 mL, *P* = .004) compared with those with shorter episodes (Table 6). Among patients with high-grade MRA stenosis (Grade 3), the stroke recurrence rate reached 42.9%, significantly higher than other groups (*P* = .009, Table 7).

**Table 6:** Imaging findings and outcomes stratified by symptom duration.

Symptom duration	n	DWI positive, n (%)	Median hypoperfusion volume, mL (IQR)	90-d stroke, n (%)
<1 h	62	16 (25.8)	9.2 (5.4–16.3)	7 (11.3)
≥1 h	44	21 (47.7)	16.8 (9.7–25.1)	9 (20.5)
*P*-value	–	.012	.004	.144

DWI = diffusion-weighted imaging.

**Table 7:** Ninety-day stroke rates by MRA stenosis grade

MRA grade	Total n	90-d stroke, n (%)	*P*-value (vs grade 0)
Grade 0 (no stenosis)	55	5 (9.1)	Reference
Grade 1 (<50%)	24	3 (12.5)	.631
Grade 2 (50%–70%)	20	3 (15.0)	.447
Grade 3 (>70%)	7	3 (42.9)	.009

CI = confidence interval, IQR = interquartile range, MRA = magnetic resonance angiography, NPV = negative predictive value, PPV = positive predictive value.

## 4. Discussion

The principal finding is that the combined 3D-ASL and DWI significantly enhances the detection of cerebrovascular pathology in TIA patients and improves the prediction of 90-day stroke risk compared with either modality alone. Our data demonstrate that while DWI identified acute ischemic lesions in 34.9% of TIA patients, 3D-ASL detected cerebral hypoperfusion in 60.4% of cases, including 33% with DWI-negative findings. The combined approach achieved an AUC of 0.854 for predicting stroke recurrence, representing a clinically meaningful improvement over single-modality assessment.

DWI is the current cornerstone of TIA neuroimaging, but its well-documented limitation lies in low sensitivity (30%–45%) for detecting subtle or transient ischemic changes^[14,15]^ – consistent with our finding that DWI identified acute lesions in only 34.9% of TIA patients. In contrast, 3D-ASL detected cerebral hypoperfusion in 60.4% of cases, including 33.0% of the cohort (35 patients) who were DWI-negative. Critically, these DWI-negative/3D-ASL-positive patients had a 20.0% 90-day stroke rate, significantly higher than the 0% rate in those with concordant negative imaging (DWI-/3D-ASL-), challenging the traditional assumption that “DWI negativity equals low risk.”^[16]^ This distinction stems from their differing pathophysiological targets: DWI visualizes irreversible cytotoxic edema from established infarction, while 3D-ASL captures reversible hemodynamic impairment in the ischemic penumbra – tissue with reduced CBF but preserved viability that remains vulnerable to recurrent ischemia.^[17,18]^ In DWI-negative TIA patients, 3D-ASL-detected hypoperfusion likely reflects persistent penumbral tissue from micro-embolism or transient vessel occlusion, which induces reversible perfusion deficits without the cytotoxic edema required for DWI detection. Clinically, this subgroup warrants prioritization for intensified secondary prevention (e.g., dual antiplatelet therapy, high-intensity statins, or urgent revascularization for severe stenosis) over routine outpatient follow-up.^[19]^

Although previous studies have reported associations between 3D-ASL-detected hypoperfusion and stroke recurrence,^[20,21]^ our study advances the field by identifying a specific quantitative threshold. Hypoperfusion volume >15 mL was an independent predictor of 90-day stroke (HR 2.87, 95% CI 1.06–7.78, *P* = .038). This threshold is biologically plausible, as larger hypoperfusion volumes indicate more extensive hemodynamic compromise, which is linked to 2 key mechanisms of stroke recurrence: increased likelihood of incomplete collateral circulation (leaving tissue outside the primary ischemic zone inadequately perfused) and higher probability of underlying severe vascular pathology (e.g., tandem stenosis or unstable plaque) that elevates the risk of recurrent embolism.^[21,22]^ The moderate positive correlation between hypoperfusion volume and ADC reduction rate (*R* = 0.512, *P* = .001) further supports this mechanistic link, as expanding perfusion deficits correspond to greater extent of irreversible tissue damage.^[23]^ Notably, even in DWI-negative patients, hypoperfusion volume >15 mL was associated with higher stroke risk (17.1% vs 3.2% in those with volume ≤ 15 mL, data not shown), indicating that volume quantification adds prognostic value beyond mere qualitative assessment of hypoperfusion presence. This threshold provides clinicians with an objective tool for risk stratification: TIA patients with hypoperfusion volume >15 mL can be admitted for inpatient monitoring and aggressive risk factor management, while those with smaller volumes may be suitable for outpatient follow-up with close surveillance.

The combined 3D-ASL plus DWI approach achieved an AUC of 0.854 for 90-day stroke prediction, significantly outperforming DWI alone (AUC 0.761) and 3D-ASL alone (AUC 0.790) (both *P* <.05). This superior performance reflects integration of 2 distinct but complementary pathophysiological pathways: DWI identifies established ischemic damage (a marker of recent severe ischemia), whereas 3D-ASL identifies persistent hemodynamic impairment (a marker of ongoing vulnerability).^[24,25]^ Stratification by imaging patterns further clarifies risk gradients: patients with dual-positive findings (DWI+/ASL+) had the highest stroke rate (31.0%), followed by DWI-/ASL + (20.0%), DWI+/ASL- (12.5%), and DWI-/ASL- (0%). This gradient aligns with the “ischemia severity continuum,” where DWI+/ASL + indicates both irreversible infarction and surrounding hypoperfused penumbra (the most severe and high-risk phenotype), DWI-/ASL + reflects isolated but significant hemodynamic compromise, DWI+/ASL- may indicate small, transient infarctions with restored perfusion (lower recurrence risk), and DWI-/ASL- suggests minimal or no significant ischemic insult.^[11]^ This stratification enables personalized intervention: dual-positive patients may require urgent vascular imaging (e.g., carotid duplex or CTA) and potential revascularization, while DWI-/ASL + patients need intensified medical therapy, and dual-negative patients can be managed with standard secondary prevention.

Multivariate analysis confirmed that imaging parameters (DWI positivity, hypoperfusion volume >15 mL) and the ABCD^2^ score ≥4 (HR = 3.01, 95% CI:1.09–8.31, *P* = .033) are independent prognostic factors – this underscores that clinical scoring and multimodal imaging capture distinct dimensions of stroke risk. The ABCD^2^ score, as a widely used bedside tool, efficiently stratifies risk via readily available clinical variables (age, blood pressure, symptom duration), but its inherent limitation lies in inability to detect subclinical hemodynamic impairment or subtle vascular pathology.^[26]^ For instance, 12.3% of our patients with ABCD^2^ score 0 to 3 (traditionally “low-risk”) were 3D-ASL-positive, and 16.7% of these developed stroke within 90 days – this subgroup would be undertreated without imaging supplementation. Conversely, among patients with ABCD^2^ score ≥4 (high-risk), 28.9% had concordant DWI-/ASL- findings and no stroke recurrence, indicating that clinical scoring alone may overestimate risk in some cases.

Our findings build on and extend previous research in several key ways. First, while prior studies have reported that 3D-ASL improves TIA risk stratification,^[27,28]^ none have established a specific hypoperfusion volume threshold for stroke recurrence. Our identification of 15 mL as an independent prognostic factor provides an actionable, objective biomarker that can be integrated into clinical workflows. Second, most studies have focused on the additive value of 3D-ASL to DWI, but few have analyzed stroke risk across all 4 imaging patterns (DWI+/ASL+, DWI+/ASL-, DWI-/ASL+, DWI-/ASL-). Our data clarify the risk gradient across these phenotypes, enabling more precise patient triage. Third, we demonstrate that symptom duration ≥1 hour correlates with higher DWI positivity (47.7% vs 25.8%, *P* = .012) and larger hypoperfusion volumes (16.8 vs 9.2 mL, *P* = .004) – a finding that helps clinicians prioritize imaging resources. Patients with longer symptoms are more likely to have detectable ischemia, justifying urgent MRI, while those with very brief symptoms (<1 hour) may have lower imaging yield but still require assessment if clinical risk factors are present. Notably, our 3D-ASL hypoperfusion detection rate (60.4%) aligns with a study by Qiao et al,^[29]^ which reported a 55.8% detection rate of perfusion deficits via ASL in DWI-negative TIA patients – supporting the reproducibility of our findings.

Despite its strengths, this study has several limitations that should be addressed in future research. First, the single-center, retrospective design may limit generalizability, as the cohort may not be representative of community-based TIA patients (e.g., older adults with more comorbidities or patients with limited access to MRI). Multicenter prospective studies are needed to validate the 15 mL hypoperfusion threshold and imaging pattern risk stratification in diverse populations. Second, we did not perform a head-to-head ROC analysis of the “3D-ASL + DWI + ABCD^2^” model vs. the “ABCD^2^ alone” model, which would quantify the incremental prognostic value of imaging. Future studies should address this gap to determine whether adding 3D-ASL to clinical scoring significantly improves risk prediction beyond ABCD^2^ alone. Third, we did not collect data on specific secondary prevention strategies (e.g., type of antiplatelet therapy, statin dosage, compliance) or detailed vascular imaging results (e.g., carotid stenosis severity, plaque morphology), which may influence stroke recurrence rates. Future studies should integrate these variables to adjust for confounding and clarify the interaction between treatment intensity and imaging findings. Fourth, the number of patients with high-grade MRA stenosis (Grade 3, n = 7) was small, limiting our ability to analyze the interaction between vascular stenosis, hypoperfusion volume, and stroke risk. Larger cohorts with more patients with severe stenosis are needed to explore this relationship. Fifth, 3D-ASL acquisition parameters (e.g., post-labeling delays, TR/TE) vary across MRI scanners, which may affect hypoperfusion volume quantification. Standardization of 3D-ASL protocols and external validation of the 15 mL threshold across different scanner platforms are necessary for widespread clinical adoption. In additional, while all MRI examinations were performed within 72 hours of symptom onset as per our inclusion criteria, we did not systematically record the exact interval from symptom onset to imaging acquisition. This represents a notable limitation given the dynamic evolution of ischemic changes in the acute phase. Consequently, patients imaged at 12 hours versus 60 hours may exhibit substantially different hypoperfusion volumes despite similar underlying pathophysiology. Our reported >15 mL threshold should therefore be interpreted with caution in the context of delayed imaging (>24–48 hours), where volumes may be underestimated.

Future research should also focus on several key areas: exploring the cost-effectiveness of routine 3D-ASL in TIA workups to determine whether the reduction in stroke events and associated healthcare costs justifies the added imaging expense; integrating 3D-ASL parameters into machine learning models that combine clinical, imaging, and laboratory data (e.g., inflammatory markers, genetic risk factors) for personalized risk prediction; and evaluating whether targeted interventions (e.g., dual antiplatelet therapy, revascularization) in high-risk imaging subgroups (DWI+/ASL+, hypoperfusion volume >15 mL) reduce stroke recurrence rates.

## 5. Conclusion

Combining 3D-ASL with DWI substantially improves detection of cerebrovascular pathology in TIA patients and enhances 90-day stroke risk prediction. The identification of high-risk patients through this multimodal approach may enable more precise risk stratification and guide targeted therapeutic interventions. These findings support consideration of expanded perfusion imaging in the routine evaluation of TIA patients.

## Author contributions

**Conceptualization:** Yanmei Fu, Tongyu Shang, Peiqi Wang, Chuan Peng.

**Data curation:** Yanmei Fu, Tongyu Shang, Peiqi Wang, Chuan Peng.

**Funding acquisition:** Chuan Peng.

**Investigation:** Tongyu Shang, Chuan Peng.

**Methodology:** Yanmei Fu, Peiqi Wang.

**Resources:** Peiqi Wang.

**Software:** Yanmei Fu, Tongyu Shang.

**Supervision:** Tongyu Shang.

**Validation:** Yanmei Fu, Chuan Peng.

**Visualization:** Yanmei Fu, Peiqi Wang.

**Writing – original draft:** Yanmei Fu, Tongyu Shang, Chuan Peng.

**Writing – review & editing:** Chuan Peng.
